# Mediation of the Association Between Physical Exercise and Depressive/Anxiety Symptoms by Pain and Sleep Problems Among Older Adults

**DOI:** 10.1177/23337214241241397

**Published:** 2024-03-23

**Authors:** Namkee G. Choi, Bryan Y. Choi, C. Nathan Marti

**Affiliations:** 1 University of Texas at Austin, Austin, TX, USA; 2 Philadelphia College of Osteopathic Medicine and BayHealth, Dover, DE, USA

**Keywords:** depression, anxiety, physical exercise, pain, sleep, older adults

## Abstract

In this study, based on the 2022 National Health and Aging Trend Study (*N* = 5,593, age 65+), we examined direct associations between moderate and vigorous physical exercise (PE) and depressive/anxiety symptoms as well as bothersome pain and sleep problems. We then examined if the association between PE and depressive/anxiety symptoms would be partially mediated by the effects of PE on bothersome pain and sleep problems. Results from a path model showed that controlling for sociodemographic and health statuses, PE was negatively associated with depressive/anxiety symptoms and bothersome pain, but it was not significantly associated with sleep problems. The mediation analysis showed that 10% of the total effects of PE on depressive/anxiety symptoms was indirect effects of PE on bothersome pain. This study is important as it examined the associations among PE, pain, sleep, and depression/anxiety in community-dwelling older adults in their natural environments. Healthcare and social service providers for older adults need to emphasize the importance and benefits of PE for older adults’ physical and mental health. Easy access to venues for PE is also important.

## Introduction

Extensive research has shown that physical exercise (PE) interventions, including those at low-intensity levels, are effective for reducing late-life depressive symptoms (Bigarella et al., 2022; Catalan-Matamoros et al., 2016; Klil-Drori et al., 2020; Pérez-López et al., 2017; Rhyner & Watts, 2016; Schuch et al., 2016) and preventing depression in older adults (Hu et al., 2020). Studies show that PE interventions are also effective as an add-on to antidepressant medications (Murri et al., 2018), although their effects compared to antidepressant medications need further examination (Hidalgo et al., 2021; Hidalgo & DEP-EXERCISE Group, 2019). While research on PE’s effects on late-life anxiety symptoms has not been as extensive as its effects on depression, meta-analyses showed low-to-moderate intensity PE leading to alleviation of symptoms of mild-moderate anxiety in older adults (Chong et al., 2022; Loh et al., 2019; Martínez-Domínguez et al., 2018). Other studies of adults of all ages also found that PE had moderate positive effects on reducing anxiety symptoms (Kandola et al., 2019; Stubbs et al., 2017).

Research has shown that PE’s positive effects on late-life depression extend to positive impacts on cognitive function, disability, and cardiovascular health (Contreras-Osorio et al., 2022; Dotson et al., 2021; Neviani et al., 2017; Toni et al., 2016). PE is also an effective intervention for alleviating pain to a small-to-moderate degree and sleep problems in older adults (Geneen et al., 2017; Laredo-Aguilera et al., 2018; Passos et al., 2011; Reid et al., 2010). Research has also shown the significant associations of depression/anxiety symptoms with pain and sleep disturbances—especially self-reported sleep problems—in older adults (Bao et al., 2017; Becker et al., 2017; Bergmans et al., 2021; Eslami et al., 2016; Fang et al., 2019; Li et al., 2018; Liu et al., 2021; Nadorff et al., 2018; Nielson et al., 2023). A 13-year Dutch longitudinal study on aging found that the risk of developing depressive symptoms is substantial in nondepressed participants with elevated pain at baseline and the consistent association between pain and depression afterward (Hilderink et al., 2012; Sanders et al., 2015). Pain also disturbs sleep, which leads to depression/anxiety (Nijs et al., 2018). Other studies showed that those with more severe or comorbid depression/anxiety had the lowest level of physical activity or fragmentation of activity rhythm and the most circadian rhythm disruptions, pointing out the need for PE interventions for those with depression/anxiety and sleep problems (Difrancesco et al., 2019; Luik et al., 2015). A systematic review and network meta-analysis found that most PE interventions for sleep problems in depression resulted in significantly better sleep outcomes (Brupbacher et al., 2021).

Given these findings of interconnections among depression/anxiety, pain, sleep, and physical activity and PE’s positive effects on depression/anxiety, pain, and sleep, research is needed to examine if PE’s effect on depression/anxiety may be mediated by its positive effects on pain and sleep. While PE’s direct effects on depression/anxiety, pain, and sleep have been examined, little research has been done on the potential mediating effects of pain and sleep on the association between PE and depression/anxiety. Furthermore, the associations among PE, pain, sleep, and depressive/anxiety symptoms in non-experimental, real-world settings have not been examined, since most of previous studies reported on the effects of PE interventions that were tested in randomized controlled trials. Research is needed to examine the associations among PE, pain, sleep, and depression/anxiety in community-dwelling older adults in their natural environments, so that the findings have more pertinent implications in their daily life. Better understanding of these associations will be useful in designing effective real-world programs for increasing PE and reducing depression/anxiety in older adults.

In this study, using a nationally representative sample of community-dwelling Medicare beneficiaries age 65 and older, we examined if the associations between PE and depressive/anxious symptoms are mediated by the associations of PE with pain and sleep, controlling for sociodemographic and health characteristics. We chose to focus on depression/anxiety symptoms as depression and anxiety tend to be co-morbid in late life and can interact with each other to cause worse psychological and physical impacts, especially among individuals with high levels of both symptom dimensions (Beattie et al., 2010; Kircanski et al., 2017; Lenze et al., 2001). For example, in a study based on the National Comorbidity Survey-Replication, onset of anxiety disorders preceded onset of depressive disorders for more than three quarters of older adults with comorbid depression/anxiety (King-Kallimanis et al., 2009). In a study of Canadian older adults, depression was the most common comorbid disorder among respondents with any of the anxiety disorders, and those with comorbid disorders reported significantly lower well-being and greater impairment than those with depression or anxiety alone (Cairney et al., 2008). More important, studies have shown that the risk factors for anxiety and depression, including sleep problem and medical conditions, overlap significantly across the life span (Beattie et al., 2010), and that those with pain and both depression and anxiety experienced the greatest pain severity and pain-related disability (Bair et al., 2008).

**Figure 1.**
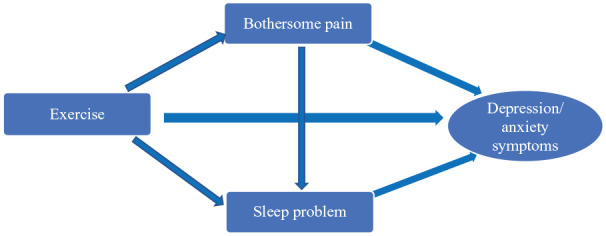
Conceptual framework for direct effects of physical exercise on depressive/anxiety symptoms. *Note.* Arrows represent: (1) direct effects of PE, bothersome pain, and sleep problems on depressive/anxiety symptoms; (2) direct effects of PE on bothersome pain and on sleep problems; and (3) direct effects of bothersome pain on sleep problems. Indirect effects of PE on depressive/anxiety symptoms through bothersome pain and sleep problems are respectively the product of bothersome pain regressed on PE × depressive/anxiety symptoms regressed on bothersome pain and sleep problems regressed on PE × depressive/anxiety symptoms regressed on sleep problems.

Based on previous study findings, study hypotheses were: (H1) depressive/anxiety symptoms would be negatively associated with moderate and vigorous PE but positively associated bothersome pain and sleep problems; (H2) both bothersome pain and sleep problems will be negatively associated with PE; and (H3) the association between PE and depressive/anxiety symptoms would be partially mediated by the effects of PE on bothersome pain and sleep problems. Please refer to Figure 1 for conceptual/analytic framework. The study findings will provide added insights into the associations among PE, pain, sleep problems, and depressive/anxiety symptoms among community-dwelling older adults in their natural environments.

## Methods

### Data and Sample

Data came from the 2022 U.S. National Health and Aging Trends Study (NHATS). NHATS collects data annually from a nationally representative panel of Medicare beneficiaries on participants’ physical, functional, cognitive, and sensory capacity, social, physical, and technological environments, and participation in valued activities. The initial NHATS sample persons (age 65+) were first interviewed in 2011, and the first and second replenishment samples were added in 2015 and 2022, respectively (Freedman et al., 2023). The 2022 NHATS data were collected in in-person interviews. In this study, we focused on 5,593 sample persons, representing approximately 49.4 million Medicare beneficiaries age 65+ who were living in their own homes or residential care communities (but not in nursing homes) and self-reported data (i.e., no proxy interview). We excluded proxy-interviewed sample persons (*n* = 241) to ensure that depressive/anxiety symptoms, pain, and sleep problems were self-reported. This study, based on de-identified public-use data, was exempt from the authors’ institutional review board review.

### Measures

*Past-month depression/anxiety symptoms*: In NHATS, depression/anxiety symptoms during the last month were assessed with the Patient Health Questionnaire-4 (PHQ-4) (Kroenke et al., 2009; Löwe et al., 2010). The PHQ-4 includes the first two items (PHQ-2) from the 9-item PHQ-9 for depression (Kroenke et al., 2003) and the first two items (GAD-2) from the 7-item Generalized Anxiety Disorder Scale (Spitzer et al., 2006). The PHQ-2 captures cognitive/affective symptoms of anhedonia and depressed mood: (a) had little interest or pleasure in doing things; and (b) felt down, depressed, or hopeless. The GAD-2 represents the core anxiety symptoms: (a) felt nervous, anxious, or on edge; and (b) have been unable to stop or control worrying. Responses to each PHQ-4 item were based on a 4-point scale (0 = not at all; 1 = several days; 2 = more than half the days; 3 = nearly every day), with the total score ranging from 0 to 12. Scores 0 to 2 indicating normal, 3 to 5 indicating mild, and 6 to 12 indicating moderate/severe symptoms (Kroenke et al., 2009). Unweighted Cronbach’s alpha for the PHQ-4 for the study sample was .74.

*Past-month PE*: NHATS includes two exercise variables: (1) ever went walking for exercise (yes or no), and (2) ever spent time on vigorous activities that increased heart rate or made breathe harder, for example, working out, swimming, running or biking, or playing a sport (yes or no). In this study, we created a dichotomous PE variable: any exercise (ever walked and/or spent time on vigorous activity = 1) versus not engaged in any walking for exercise or vigorous activity = 0). Previous research has shown that most types of PE, such as walking and aerobics, had positive effects on depression/anxiety, pain, and sleep (Brupbacher et al., 2021; Schuch et al., 2016; Suh et al., 2019). However, for sensitivity analysis, we also created a 4-category PE variable (not engaged in any walking for exercise or vigorous activity = 0; walked but did not do any vigorous activity = 1; engaged in vigorous activity but did not walk = 3; engaged in walking and vigorous activity = 4) to examine whether or not these types of PE may be differentially associated with depression/anxiety, bothersome pain, and sleep problems.

*Past-month bothersome pain*: Sample persons were asked if they were bothered by pain in the past month (yes = 1, no = 0).

*Past-month sleep problem*: NHATS includes three questions about sleep: how often it took more than 30 min to fall asleep at night, how often respondents had trouble falling back asleep on nights when woken up, and how often they took medication to sleep. Response categories were: every night, most nights, some nights, rarely, never. In this study, we defined a sleep problem as taking more than 30 min to fall asleep, trouble falling back asleep, or taking sleep medication every night, most nights, or some nights (yes = 1, no = 0)

*Health status*: This included: (1) number of diagnosed chronic conditions (0–9; arthritis, cancer, diabetes, heart attack or heart disease, hypertension, lung disease, osteoporosis, stroke, and other serious illness); (2) self-reported diagnosis of dementia; and (3) self-reported mobility help within the past year or more (derived from the question: “Have you been getting help with getting out of bed, getting around your home/building, leaving your home/building for a year or more?”). To be conservative, any missing data for the study variables were treated as “0” or not having the condition.

*Sociodemographic factors*: These were age in 2022 (65–69, 70–74, 75–79, 80–84, 85–89, 90+; under 80 vs. 80 and older in multivariable analysis); gender (female vs. male); race/ethnicity (non-Hispanic White, non-Hispanic Black, Hispanic, all other); marital status (married/partnered, divorced/separated, widowed, never married); and education (bachelor’s degree vs. no degree).

### Analysis

All analyses were conducted with Stata/MP 18’s svy function (College Station, TX) to account for NHATS’s stratified, multistage sampling design (Jiao et al., 2023). First, we presented univariate frequencies for the study population characteristics. Second, we fitted a path model for hypotheses testing (direct effect of PE, pain, and sleep problems on depression/anxiety symptoms and the mediation effects of pain and sleep problems on the association between PE and depression/anxiety symptoms). Results for direct and mediation effects are reported as coefficients and linearized standard errors with 95% confidence intervals (CI). To test the statistical significance of indirect effects of two mediators (pain and sleep problems), we used bootstrapped (10,000 repetitions) analysis. We calculated the ratio of the indirect effects to the total effects (direct effect of PE on depressive/anxiety symptoms + indirect effects of the mediators). For sensitivity analyses, we used generalized path models (with gsem command in Stata) to test: (1) the 3-categorization (normal, mild, and moderate/severe) of depressive/anxiety symptoms; and (2) the 4-categorization of PE (no PE, walking only, vigorous activity only, both walking and vigorous activity). The results from these generalized path models did not significantly deviate from those of the path model that included the depression/anxiety symptoms as a continuous variable and PE as a dichotomous variable. Thus, we presented the results from the latter path model only.

## Results

### Characteristics of the Study Population

Table 1 shows that 10% of the study population were 80 years and older, 22% were Black, Hispanic, or Other, 37% had a bachelor’s degree or higher, and 96% lived in their own home. They had, on average, 2.6 (*SE* = 0.03) chronic and/or other serious medical conditions (not counting dementia), 2.6% reported a diagnosis of dementia, and 7.6% reported that they had help with mobility inside and/or outside their home during the past year. Seventy-seven percent engaged in PE in the preceding month, and 56% and 66% reported bothersome pain and sleep problems, respectively. Of those who engaged in PE, 89% reported walking, with or without also engaging in vigorous activities, indicating that 69% of the study population reported walking for exercise in the past month. The average depressive/anxiety symptom score was 1.7 (*SE* = 0.03), and 8% had moderate/severe symptoms.

**Table 1.** Sample Characteristics.

Characteristic	% or *M* (*SE*)
Age group (years, %)	
65–69	26.8
70–74	30.1
75–79	21.5
80–84	11.7
85–89	6.6
90+	3.3
Gender (%)	
Female	54.8
Male	45.2
Race/ethnicity (%)	
Non-Hispanic white	78.3
Black	8.6
Hispanic	8.2
Other	4.9
Marital status (%)	
Married/co-habiting	59.1
Divorced/separated	15.2
Widowed	21.3
Never married	4.4
Education (%)	
<Bachelor’s degree	62.9
≥Bachelor’s degree	37.1
Residence (%)	
Own home	96.1
Care community	3.9
No. of medical condition, *M* (*SE*)	2.55 (0.03)
Dementia diagnosis (%)	2.6
Past-year mobility help (%)	7.5
Past-month exercise (%)	76.9
Walking only	30.8
Vigorous activity only	8.3
Both walking and vigorous activity	37.8
Past-month bothersome pain (%)	55.7
Past-month sleep problem (%)	65.5
Past-month depression/anxiety symptoms, *M* (*SE*)	1.73 (0.05)
Depression/anxiety symptom group (%)	
None (0–2)	73.3
Mild symptoms (3–5)	18.6
Moderate/severe (6–12)	8.1

### Direct Effects of PE, Bothersome Pain, and Sleep Problems

Table 2 shows the mediation model results, that is, direct effects of PE, bothersome pain, and sleep problems on depression/anxiety symptoms and the effects of PE on bothersome pain and sleep problems. The top rows (direct effects) show that, as hypothesized, depressive/anxiety symptoms were negatively associated with PE (*B* = −0.54, *SE* = 0.11, *t* = −5.05, *p* < .001), but positively associated with bothersome pain (*B* = 0.78, *SE* = 0.08, *t* = 9.62, *p* < .001) and sleep problems (*B* = 0.85, *SE* = 0.09, *t* = 9.23, *p* < .001). Results from our sensitivity analyses in which we used three categories of depressive/anxiety symptoms (none, mild, moderate/severe) and four categories of PE (no PE, walking only, vigorous activity only, both walking ad vigorous activity) did not significantly deviate from these results. The findings support H1. Depressive/anxiety symptoms were also positively associated with female gender, divorced state, number of medical conditions, dementia diagnosis, and the receipt of mobility help, but negatively associated with age 80 and older.

**Table 2.** Effects of Physical Exercise, Bothersome Pain, and Sleep Disturbance on Depression/Anxiety Symptoms: Model Parameters From Mediation Model Using Structural Equation Modeling.

Model parameter	*B* (*SE*)	*t*	*p*	95% CI
Depression/anxiety score				
PE	–0.54 (0.11)	–5.05	<.001	[–0.75, –0.32]
Bothersome pain	0.78 (0.08)	9.62	<.001	[0.62, 0.94]
Sleep problem	0.85 (0.09)	9.23	<.001	[0.67,1.04]
Age 80 and older	–0.32 (0.08)	–4.17	<.001	[–0.48, –0.17]
Female	0.25 (0.10)	2.48	.016	[0.05, 0.45]
Black	–0.07 (0.13)	–0.54	.592	[–0.34, 0.19]
Hispanic	0.22 (0.13)	1.66	.102	[–0.04, 0.47]
Other race/ethnicity	–0.06 (0.18)	–0.30	.763	[–0.42, 0.31]
Divorced	0.49 (0.15)	3.24	.002	[0.19, 0.80]
Widowed	0.10 (0.11)	0.85	.401	[–0.13, 0.33]
Never married	0.21 (0.20)	1.05	.299	[–0.19, 0.61]
≥Bachelor’s degree	–0.14 (0.10)	–1.43	.159	[–0.34, 0.06]
No. of medical conditions	0.16 (0.03)	5.89	<.001	[0.11, 0.21]
Dementia diagnosis	1.21 (0.25)	4.84	<.001	[0.71, 1.71]
Mobility help	1.19 (0.15)	7.77	<.001	[0.89, 1.50]
Bothersome pain				
PE	–0.07 (0.02)	–3.90	<.001	[–0.11, –0.04]
Age 80 and older	–0.04 (0.02)	–2.84	.006	[–0.07, –0.01]
Female	0.03 (0.02)	1.22	.229	[–0.02, 0.08]
Black	–0.03 (0.02)	1.26	.212	[–0.07, 0.02]
Hispanic	–0.04 (0.02)	–1.85	.069	[–0.09, 0.00]
Other race/ethnicity	–0.16 (0.04)	–3.83	<.001	[–0.24, –0.07]
Divorced	0.05 (0.03)	1.87	.066	[–0.00, 0.10]
Widowed	–0.01 (0.02)	–0.65	.520	[–0.06, 0.03]
Never married	–0.00 (0.04)	–0.03	.977	[–0.09, 0.08]
≥Bachelor’s degree	–0.00 (0.02)	–0.23	.823	[–0.05, 0.04]
No. of medical conditions	0.09 (0.01)	14.97	<.001	[0.08, 0.10]
Dementia diagnosis	–0.05 (0.05)	–1.01	.318	[–0.14, 0.05]
Mobility help	0.13 (0.03)	3.91	<.001	[0.06, 0.19]
Sleep problem				
PE	0.01 (0.02)	0.69	.490	[–0.03, 0.05]
Bothersome pain	0.12 (0.02)	5.64	<.001	[0.08, 0.16]
Age 80 and older	–0.00 (0.01)	–0.18	.855	[–0.03, 0.02]
Female	0.05 (0.02)	2.33	.024	[0.01, 0.09]
Black	–0.00 (0.02)	–0.16	.873	[–0.05, 0.04]
Hispanic	–0.02 (0.02)	–0.73	.467	[–0.06, 0.03]
Other race/ethnicity	0.07 (0.04)	1.53	.133	[–0.02, 0.15]
Divorced	0.04 (0.03)	1.43	.158	[–0.02, 0.09]
Widowed	0.03 (0.02)	1.28	.207	[–0.02, 0.08]
Never married	–0.02 (0.05)	–0.49	.625	[–0.13, 0.08]
≥Bachelor’s degree	–0.04 (0.02)	1.67	.101	[–0.09, 0.01]
No. of medical conditions	0.03 (0.00)	6.07	<.001	[0.02, 0.04]
Dementia diagnosis	0.10 (0.04)	2.42	.019	[0.02, 0.17]
Mobility help	0.08 (0.03)	2.71	.009	[0.02, 0.14]

*Note. N* = 5,593; Population size = 49,362,716; Number of strata = 56; Number of PSUs = 112, Design *df* = 56.

Reference group for Black, Hispanic, and Other was non-Hispanic White. Reference group for divorced, widowed, and never married was married/co-habiting.

The middle and bottom rows in Table 2 show that controlling for sociodemographic and health statuses, PE was negatively associated with bothersome pain (*B* = −0.07, *SE* = 0.02, *t* = −3.90, *p* < .001), but not significantly associated with sleep problems (*B* = 0.01, *SE* = 0.02, *t* = 0.69, *p* = .490), although bothersome pain was positively associated with sleep problems (*B* = 0.12, *SE* = 0.02, *t* = 5.64, *p* < .001). Bootstrapped results show a significant indirect effect (−0.06, 95% CI = [−0.09, −0.02], *z* = −3.18, *p* = .001) of PE on depressive/anxiety symptoms through bothersome pain, but no significant indirect effect (0.01, 95% CI = [−0.02, 0.09], *z* = 5.05, *p* = .67) of PE on depressive/anxiety symptoms through sleep problems. Again, the results from our sensitivity analyses in which we used four categories of PE did not significantly deviate from these results. One deviation was that vigorous activity was not significantly associated with bothersome pain; however, walking with or without vigorous activity was. The ratio of indirect effect of bothersome pain on PE (−0.06) to the total effect of PE on depressive/anxiety symptoms (indirect effect [−0.06] + direct effect [−0.54] = −0.59) were 0.10. These results partially support the second and third hypothesis.

## Discussion and Conclusion

Along with many other health benefits (Izquierdo et al., 2021; Northey et al., 2018), evidence of PE’s positive effects on depressive/anxiety symptoms is well-established (Bigarella et al., 2022; Catalan-Matamoros et al., 2016; Chong et al., 2022; Martínez-Domínguez et al., 2018). On the other hand, pain and sleep problems are known risk factors for late-life depression (Nielson et al., 2023; Schaakxs et al., 2017). Since PE has also been shown to alleviate pain and sleep problems in older adults, we hypothesized that PE’s effects on depression/anxiety symptoms would be partially mediated through its effects on pain and sleep problems among a nationally representative sample of community-dwelling Medicare beneficiaries age 65 and older. Our results confirmed the associations between PE and lower depressive/anxiety symptoms, regardless of the types of PE, and the associations between both bothersome pain and sleep problems and higher depressive/anxiety symptoms. The results also confirmed that the effect of PE on depressive/anxiety symptoms is partially (10% of total effects) mediated by PE’s effect on bothersome pain. However, PE did not have positive effects on sleep problems, thus, no mediation effects via sleep problems on depressive/anxiety symptoms, pointing to the need for further studies of PE and sleep problems in older adults.

The significant associations between PE and pain and depression/anxiety among older adults in their natural environments confirms PE’s beneficial physical and mental health effects that have already been established in experimental studies as described earlier. Our findings add to the existing knowledge base especially given that the majority of older adults in the study engaged in walking, and that walking, with or without vigorous activity engagement, was significantly inversely associated with bothersome pain. The inverse association between PT and depressive/anxiety symptoms did not vary by the types of PE, either. Compared to some other types of PE, walking (both inside and outside one’s dwelling) can be relatively easily incorporated in daily routines of older adults without special equipment. Multifaceted benefits of walking for healthy aging has been discussed in other studies (Tomata et al., 2019; Ungvari et al., 2023). Our findings are also in line with previous systematic reviews and meta-analysis studies that found the effectiveness of low-to-moderate intensity PE for depression/anxiety and pain treatment (Chong et al., 2022; Schuch et al., 2016; Wen et al., 2022).

One of the reasons for the lack of association between PE and sleep problems may be attributed to the lack of reliability and validity of self-reports of sleep problems and the lack of data on PE frequency and duration. A systematic review found that moderate intensity exercise programs, with a frequency of three times per week and a duration of 12 weeks up to 6 months, showed the highest number of significant improvements in different sleep outcomes in older adults (Vanderlinden et al., 2020). International PE guidelines for older adults also emphasize PE’s dose-response effects (Izquierdo et al., 2021). Another potential explanation for the lack of association between PE and sleep problems may be because the relationship is more complex than that between PE and bothersome pain. Sleep disorders in older adults increase with aging, likely due to increased sleep latency, decreased sleep efficiency, and total sleep time, and they are impacted by medical, psychiatric, environmental, and psychosocial factors (Cohen et al., 2022; Jaqua et al., 2023). Thus, in addition to PE, other nonpharmacological treatments, such as cognitive behavioral therapy, sleep hygiene education, relaxation therapy, sleep restriction, light therapy, and stimulus control therapy, may be needed for older adults (Brewster et al., 2022; Jaqua et al., 2023).

There are some study limitations due to data constraints: (1) only correlations, not causations, can be derived from cross-sectional survey data; (2) data on depression/anxiety, bothersome pain, and sleep problems were self-reported and may have been subjected to recall and social desirability bias; and (3) bothersome pain was measured with one item with a “yes or no” response, and did not include pain severity. More research is needed to further clarify the indirect effects of PE on depressive/anxiety symptoms via pain severity and sleep problems accounting for often bidirectional associations of pain and sleep problems in older adults (Chen et al., 2019).

Despite these limitations, the findings confirm the significance of PE (including walking) for alleviating pain and depressive/anxiety problems in older adults. Even though PE frequency and duration were not factored in, PE’s significant inverse association with depression/anxiety and bothersome pain in community-residing older adults need to be widely disseminated to encourage older adults to engage in PE. Most older adults are aware of the benefits of physical activity, but report a range of barriers to physical activity participation (Franco et al., 2015). Older adults prefer non-opioid analgesics and nonpharmacological interventions such as prayer/meditation, and exercise for pain treatment (Robinson-Lane & Vallerand, 2018). In addition to pain and depression/anxiety, PE’s positive effect on a wide range of health problems, functional improvement, fall and fall injury prevention, and dementia prevention (Choi et al., 2023; De la Rosa et al., 2020; Huang et al., 2022; Izquierdo et al., 2021; Rikkonen et al., 2023) need to be disseminated. Our findings show that walking appears to as good as vigorous activity and walking-based PE should be an important strategy for promoting healthy aging. Healthcare and social service providers need to emphasize the importance and benefits of PE for older adults’ physical and mental health and assist them to improve exercise habits and utilize any exercise programs available in the community. Easy access to walkable environments and other PE venues in the neighborhoods is also needed for all community-dwelling older adults. Where such venues are available in the community, older adults need to be encouraged to come to the venues, participate in PE, and reap the benefits and any financial access barriers to physical activity opportunities need to be removed.
